# Assessing the current state of ecological connectivity in a large marine protected area system

**DOI:** 10.1111/cobi.13580

**Published:** 2020-09-05

**Authors:** Kelsey E. Roberts, Carly N. Cook, Jutta Beher, Eric A. Treml

**Affiliations:** ^1^ School of Marine and Atmospheric Sciences Stony Brook University, Stony Brook New York; ^2^ School of Biological Sciences Monash University Clayton Victoria Australia; ^3^ School of BioSciences The University of Melbourne Melbourne Victoria Australia; ^4^ School of Life and Environmental Sciences, Centre for Integrative Ecology Deakin University Geelong Victoria Australia

**Keywords:** biodiversity conservation, ecoregions, larval dispersal, Great Barrier Reef, marine spatial planning, network analysis, análisis de redes, conservación de la biodiversidad, dispersión larval, ecoregiones, Gran Barrera de Arrecife, planeación espacial marina, 生物多样性保护, 生态区, 幼体扩散, 大堡礁, 海洋空间规划, 网络分析

## Abstract

The establishment of marine protected areas (MPAs) is a critical step in ensuring the continued persistence of marine biodiversity. Although the area protected in MPAs is growing, the movement of individuals (or larvae) among MPAs, termed connectivity, has only recently been included as an objective of many MPAs. As such, assessing connectivity is often neglected or oversimplified in the planning process. For promoting population persistence, it is important to ensure that protected areas in a system are functionally connected through dispersal or adult movement. We devised a multi‐species model of larval dispersal for the Australian marine environment to evaluate how much local scale connectivity is protected in MPAs and determine whether the extensive system of MPAs truly functions as a network. We focused on non‐migratory species with simplified larval behaviors (i.e., passive larval dispersal) (e.g., no explicit vertical migration) as an illustration. Of all the MPAs analyzed (approximately 2.7 million km^2^), outside the Great Barrier Reef and Ningaloo Reef, <50% of MPAs (46‐80% of total MPA area depending on the species considered) were functionally connected. Our results suggest that Australia's MPA system cannot be referred to as a single network, but rather a collection of numerous smaller networks delineated by natural breaks in the connectivity of reef habitat. Depending on the dispersal capacity of the taxa of interest, there may be between 25 and 47 individual ecological networks distributed across the Australian marine environment. The need to first assess the underlying natural connectivity of a study system prior to implementing new MPAs represents a key research priority for strategically enlarging MPA networks. Our findings highlight the benefits of integrating multi‐species connectivity into conservation planning to identify opportunities to better incorporate connectivity into the design of MPA systems and thus to increase their capacity to support long‐term, sustainable biodiversity outcomes.

## Abstract 2

*Article impact statement*: Numerous marine protected areas around Australia are not performing as a functionally connected network.

## Abstract (es)

Valoración del Estado Actual de la Conectividad Ecológica en un Sistema Extenso de Áreas Marinas Protegidas

**Resumen**

La creación de áreas marinas protegidas (AMP) es un paso muy importante para asegurar la persistencia de la biodiversidad marina. Aunque el área protegida dentro de las AMP está creciendo, el movimiento de individuos (o larvas) entre las AMP, denominado conectividad, sólo ha sido incluido recientemente como un objetivo para muchas AMP. Por lo anterior es normal que con frecuencia se ignora la evaluación de la conectividad o se sobresimplifica durante el proceso de planeación. Para promover la persistencia poblacional es importante asegurar que las áreas protegidas en un sistema estén conectadas funcionalmente por medio de la dispersión o el movimiento de individuos adultos. Diseñamos un modelo multiespecie de la dispersión larval para el ambiente marino australiano y así evaluar cuán protegida está la conectividad a escala local en las AMP y determinar si el sistema extensivo de AMP realmente funciona como una red. Nuestro diseño se enfocó en especies no migratorias con comportamientos larvales simplificados (es decir, dispersión larval pasiva) (p. ej.: sin migración vertical explícita) como un ejemplo. De todas las AMP analizadas (aproximadamente 2.7 millones de km^2^), fuera de la Gran Barrera de Arrecifes y el Arrecife Ningaloo, <50% de las AMP (46‐80% del área total de la MPA dependiendo de la especie considerada) estaba conectado funcionalmente. Nuestros resultados sugieren que el sistema de AMP australiano no puede ser considerado como una sola red sino más bien como una colección de numerosas redes más pequeñas delineadas por interrupciones naturales en la conectividad del hábitat arrecifal. De acuerdo con la capacidad de dispersión del taxón de interés, puede haber entre 25 y 47 redes ecológicas individuales distribuidas a lo largo del ambiente marino australiano. La necesidad de primero evaluar la conectividad natural subyacente de un sistema de estudio previo a la implementación de nuevas AMP representa una prioridad de investigación clave para aumentar estratégicamente las redes de AMP. Nuestros resultados resaltan los beneficios de la integración de la conectividad multiespecie dentro de la planeación de la conservación para identificar las oportunidades que mejor incorporen la conectividad en el diseño de los sistemas de AMP y así incrementar su capacidad para soportar resultados sustentables de biodiversidad a largo plazo.

## 摘要

建立海洋保护区是确保海洋生物多样性长期续存的关键步骤。虽然海洋保护区的覆盖范围在不断扩大, 但海洋保护区之间动物个体 (或幼体) 的运动情况 (即连接度) 在最近才被纳入许多海洋保护区的保护目标之中。因此, 规划过程中往往会忽视或过度简化对连接度的评估。为了促进海洋生物的种群续存, 确保系统中的保护区可以通过生物个体扩散或成体运动来实现功能性连接是十分重要的。本研究设计了一个澳大利亚海洋环境中幼体扩散的多物种模型, 以评估海洋保护区在局域尺度的连接度保护, 并确定大范围的海洋保护区系统是否真正起到了保护网络的作用。我们聚焦于幼体行为简单的非洄游物种, 如没有明显垂直迁移的被动幼体扩散的物种, 以作为例证。在我们分析的所有海洋保护区中 (约 270 万平方公里), 位于大堡礁和宁格罗礁之外的海洋保护区只有不到 50% 是功能性连接的, 占海洋保护区总面积的 46‐80% (取决于纳入考虑的物种) 。我们的研究结果表明, 澳大利亚海洋保护区系统不能被视为一个单一的网络, 而是由许多较小的网络组成的集合, 这些网络由珊瑚礁栖息地之间的自然隔断所划分。我们基于研究关注类群的扩散能力分析得出, 澳大利亚海洋环境中可能分布着 25 到 47 个独立的生态网络。因此, 在建立新的海洋保护区之前, 应首先评估研究系统潜在的自然连接度, 这是战略性扩大海洋保护区网络的研究关键点。我们的研究结果还突出了将多物种连接度纳入保护规划的好处, 这样有助于更好地将连接度纳入海洋保护区系统的设计, 从而提高其支持长期、可持续的生物多样性保护成果的能力。**【翻译: 胡怡思; 审校: 聂永刚】**

## Introduction

In an effort to halt the global decline of marine biodiversity, conserve ecosystem function, and help promote sustainable fisheries, the establishment of marine protected areas (MPAs) has rapidly increased over the past decade. In addition to well‐documented benefits, such as increased fish biomass (Edgar et al. 2014), ecosystem restoration (Campbell et al. 2018), and the movement of adult individuals to adjacent fishing grounds (i.e., spillover effect; Buxton et al. 2013), MPAs boost resilience to many anthropogenic stressors for species exploited by commercial or recreational fisheries (Magris et al. 2018). To be effective, MPAs rely on a design process guided by systematic conservation planning principles. This process can ensure that MPAs collectively represent the species and ecosystems in need of protection in order to maximize biodiversity outcomes (Margules & Pressey 2000).

A primary objective of MPAs is to ensure species persistence through the protection of important local subpopulations; thus, protected area planning must take this goal into account. At broader, network wide scales, the persistence of the entire metapopulation depends on 2 separate mechanisms: adult replacement (i.e., self‐persistence) in the local subpopulations and persistence through connectivity among local populations in a network (i.e., network persistence) (Hastings & Botsford 2006; Burgess et al. 2014). The movement of individuals (or larvae) among protected areas, termed ecological connectivity, significantly influences persistence through dynamic processes, such as self‐recruitment and colonisation, and leads to evolutionarily significant outcomes such as the flow of adaptive genes in the face of environmental change (Hoffmann & Sgrò 2011; Matz et al. 2018; Balbar & Metaxas 2019). The exchange of individuals between distinct populations typically occurs during the larval stage for many fish and invertebrate species, which largely depends on ocean currents and larval characteristics to determine likely settlement sites (Cowen & Sponaugle 2009; Treml et al. 2015). In the context of marine spatial planning, ensuring connectivity among local populations should therefore be an important consideration rather than relying on local areas to be self‐persistent (White et al. 2010; Burgess et al. 2014).

Empirically, population connectivity is difficult to measure as it occurs at multiple spatial and temporal scales and varies with species’ life history traits (Kool & Nichol 2015). Researchers have employed a variety of techniques to quantify connectivity across broad spatial scales such as genetic parentage analysis (Herrera et al. 2016), otolith chemistry analysis (Di Franco et al. 2015), and standardized recruitment monitoring (Watson et al. 2010). These approaches require extensive sampling, are often time consuming, and require long‐term data, specialised skills, and equipment to be successful. Alternatively, biophysical models of dispersal have been developed that can estimate connectivity and account for the complexity of ecological processes involved at relatively fine spatial and temporal scales (e.g., Cowen & Sponaugle 2009; Treml & Halpin  2012). Although these models are not without caveats (Paris et al. 2007; Andrello et al. 2017), dispersal modeling is a powerful tool that can provide a mechanistic understanding of larval dispersal and connectivity for any area of interest, especially when used in conjunction with other field and empirical methods.

The long‐term success of MPAs requires that they function as a connected ecological network, rather than a collection of isolated parks, to ensure exchange of individuals between populations (Santini et al. 2016). Although collections of MPAs are often referred to as a network, the term *network* implies that individual protected areas are functionally connected through the exchange of individuals between populations (Minor & Urban 2007; Treml et al. 2008). The use of connectivity as distinct criteria in MPA design thus far has been somewhat limited in comparison with terrestrial protected areas. Connectivity has been acknowledged as an important element in protected area planning through global biodiversity targets (Balbar & Metaxas 2019; Virtanen et al. 2020). Through Aichi Target 11, the Convention on Biological Diversity (CBD) emphasises the importance of well‐connected protected areas (Secretariat of the CBD 2011). In the terrestrial realm, widely used frameworks (e.g., Zonation and Marxan), illustrated through diverse case studies, allow for a relatively straightforward application of structural connectivity in spatial prioritization (Virtanen et al. 2020). Although many MPA management plans explicitly include connectivity as an objective, there has been a significant lag in incorporating connectivity in marine spatial planning (Balbar & Metaxas 2019).

The need to consider multiple species with different dispersal capabilities, as well as the complexities of dispersal modeling, can often result in connectivity generally being oversimplified or ignored in the design of MPAs (Berumen et al. 2012; Magris et al. 2016). If a goal of protection is to create an ecologically (or functionally [we use these terms interchangeably]) connected network of protected areas, then it is important to ensure the collection of established MPAs function as a network, through the dispersal of larvae, to ensure population persistence (Krueck et al. 2017). We examined and quantified the extent to which the MPA system (collection of MPAs) in Australia accommodates connectivity. Our objectives were to quantify multi‐species connectivity among all habitat patches to map dispersal pathways (objective 1); evaluate how much local scale connectivity is protected in only those habitat patches designated as MPAs (objective 2); and map where and to what extent the collection of MPAs are functionally linked by dispersal and are therefore expected to genuinely perform as a network (objective 3). We modeled multi‐species connectivity among all reef patches throughout Australia, including those in the national system of MPAs, the world's second largest collection of MPAs, based on life history parameters from 4 dispersal phenotypes to explore a range in life history and dispersal capacities.

## Methods

### Study Area

**Figure 1:**
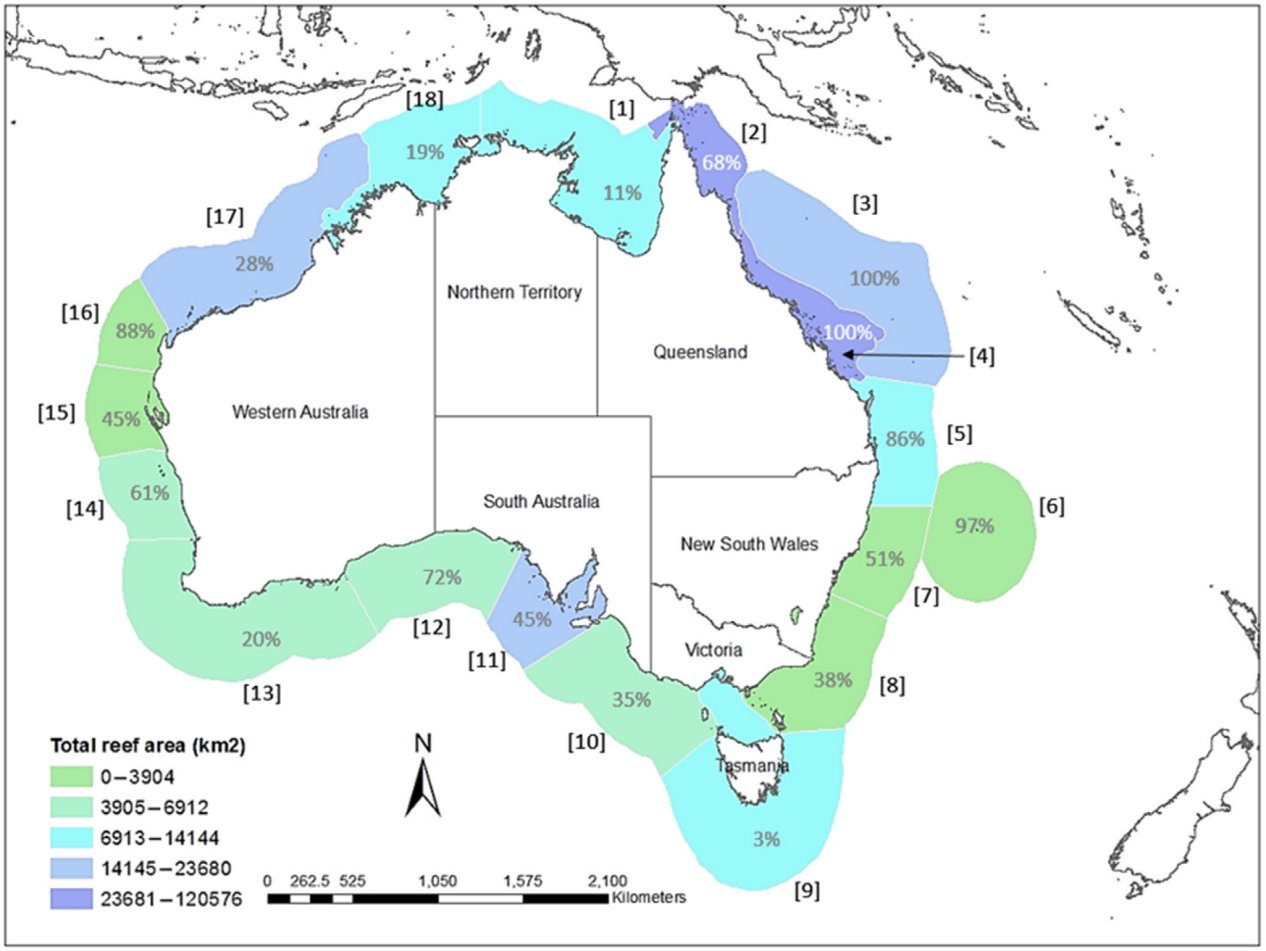
Map of the study area and surrounding ecoregions (percentages are reef in marine protected areas for each ecoregion; numbers in brackets link to ecoregion names in Table 2). Area of reefs in each ecoregion identified using natural breaks designation in GIS.

A biophysical modeling approach (Treml et al. 2012) was used to quantify potential larval dispersal between habitat patches in the Australian Exclusive Economic Zone (EEZ), excluding the Australian Antarctic Territory (Fig. 1). The model included shoreline and reef (rocky and coral) data, existing MPA boundaries, and ocean current data (∼ top 10 m [Chassignet et al. 2007]). Reef data were acquired from state and federal jurisdictions and limited to the continental shelf (up to 200 m deep). To capture the range of temporal variability in the seascape, all years of available current data were utilized (3‐hourly data for 1993–2012 at ∼0.08° resolution). A spatial resolution of 10 × 10 km was used for the biophysical model to match that of the hydrodynamic data and maintain geographic integrity while providing computational efficiencies.

The model domain of the Australian EEZ was divided into ecoregions based on the marine biogeographic classification system defined by Spalding et al. (2007) to represent ecologically distinct zones. This intermediate scale is ideally suited to visualise the geographic structure of the results across the entire EEZ at a scale appropriate for both management and further biogeographic studies. By conducting the analysis at the ecoregion scale, we accounted for geographic differences in reef presence and levels of protection, thereby creating a comparable, or normalized, illustration of the geographic patterns in connectivity. This was particularly necessary when comparing geographies outside the extensive Great Barrier Reef Marine Park, which accounts for a significant amount of Australia's reefs and protected areas and would have overwhelmed geographic patterns.

### Model Taxa

Movement between reef patches, although strongly influenced by ocean currents, is also a function of a species’ life history. The duration of the larval phase (maximum pelagic larval duration [PLD]) is a strong predictor of maximum dispersal distances (Treml et al. 2012). The time it takes for larvae to develop sufficiently to be able to settle on a substrate (i.e., the precompetency period) influences the local scale distances they travel before successfully settling in or on habitat. Together, these 2 parameters help reveal how the distance and oceanography between reefs may influence the capacity for larvae to disperse successfully to new habitats (Treml et al. 2012). Although extremely variable and largely unknown in marine species, it is important to account for daily larval mortality (e.g., due to predation, starvation), which can heavily influence dispersal potential (Treml et al. 2015). When and how often marine species release larvae (i.e., spawning window and periodicity) are also important determinants of connectivity among sites (Treml et al. 2012). Therefore, we used these important life history parameters (PLD, precompetency period, larval mortality, and spawning window) to develop dispersal phenotypes with which to model connectivity (Treml et al. 2012; Treml et al. 2015).

To parameterize the model, we sought to represent a range of dispersal phenotypes (short through long range dispersers) to account for considerations relevant to species’ ecology (i.e., body size and trophic level) and management (wide versus restricted distributions and fished versus non‐fished species). The different dispersal phenotypes were parameterized using a range of life history traits relevant to larval dispersal and compiled in a database of life history characteristics for a range of marine invertebrates and fishes. Short and intermediate range dispersers were represented by invertebrates and small reef fish with pelagic durations up to 22 days. The long range dispersers were represented by large‐bodied fish with 30 to 40 day maximum PLDs and longer precompetency periods. The 4 dispersal phenotypes (Table 1) were derived by taking the mean values of the life history parameters for approximately 10 representative species (Supporting Information [Tables S1.1–S1.4]). A 20% larval mortality per day was applied for all model simulations as a compromise between the low end (10% mortality) for large, sturdy larvae (i.e., trevally) and the high end (30% mortality) for small, fragile larvae (i.e., damselfish) (Houde 1989; Cowen et al. 2000; North et al. 2009).

**Table 1:** Input parameters used for each dispersal phenotype in ecological model of connectivity for the Australian marine environment

Dispersal phenotype and indicative taxa	Short range	Intermediate range	Long range, small bodied	Long range, large bodied
Indicative taxa	Urchin	Damselfish	Wrasse	Trevally
Life history parameters				
Maximum pelagic larval duration (days)	6	22	30	40
Precompetency period (days)	4	7	13	26
Spawning window	Oct‐Feb spawning	Oct‐Feb spawning	Annual	Annual
Larval mortality (daily %)	20	20	20	20

### Dispersal Model

Each dispersal simulation consisted of releasing a cloud of larvae, not individual particles, over a habitat patch (the quantity of larvae was proportional to the habitat area) and allowing it to be transported downstream on ocean currents according to the larval traits associated with the dispersal phenotype. This created a larval density surface (i.e., dispersal kernel), which was allowed to move throughout the seascape but was subject to biophysical parameters and solved using the fourth‐order accurate adjective transport scheme (Smolarkiewicz 2006; Treml et al. 2012). As larvae came into contact with habitat, the total number of competent larvae settling was recorded at each time‐step throughout the duration of the simulation. This was repeated for all habitat patches (284 total), for all spawning dates (1st and 15th of each month during the spawning window), for all 20 years, and for each modeled species with a high‐performance computing cluster (Lafayette et al. 2016). The computing wall‐time estimate for a single species was approximately 45 days (250 simulations per species, 4 hours per simulation). The final species‐level connectivity matrix used for all analyses was the migration matrix, where each element quantified the proportion of settlers to each destination habitat patch (column that came from each original source habitat patch [row]) and accounted for all oceanographic, habitat, and life‐history parameters (Crandall et al. 2014; Samsing et al. 2017). This migration matrix, as opposed to other matrix representations of connectivity (e.g., probability matrix), appropriately quantifies functional and demographically significant connections in the system. Despite some uncertainty in the local scale estimates near the 10 × 10 km resolution of the model, all self‐recruitment connections were initially maintained in this migration matrix. A connectivity strength threshold of 1% was then applied to the migration matrix to remove weak connections that contribute <1% of larvae to a receiving population (Andrello et al. 2017)–leaving only strong, ecologically significant connections in the analysis. Larval dispersal networks were then built for all species with the resulting matrices and habitat data (e.g., reef location, area) to analyze and visualize the connectivity patterns (objective 1).

### Model Analyses

To evaluate how much local scale connectivity is captured in the system of MPAs (objective 2), we calculated 2 local scale properties of population connectivity: rescue potential of a site, which is the degree to which the patch can be rescued by surrounding upstream source habitat patches (Kininmonth et al. 2011) and source strength, or the capacity of a site to act as a larval source to other habitat patches (Crowder et al. 2000). The rescue potential refers to the degree to which a reef patch is supported by the inflow of larvae from upstream source reefs, whereas the source strength of a habitat patch is quantified by estimating the total out flowing larvae contributing to the support of downstream reefs. Results from both measures were standardized with respect to the total inflow (for rescue potential) and outflow (for source strength) of the ecoregion of interest, allowing for an unbiased geographic representation. These metrics are sometimes referred to as dispersal flux (in or out), which represents the relative contribution of each habitat patch to surrounding patches based on the probability of dispersal and the reproductive output of the donor habitat patch (e.g., Urban & Keitt 2001). Patches with high source strength are important sources to surrounding areas and may indicate a productive habitat patch that bolsters downstream recruitment (Minor & Urban 2007). Areas of high rescue potential may receive a relatively large proportion of larvae from nearby upstream sources, which would increase resilience or recovery from disturbances and may support high levels of genetic diversity (Minor & Urban 2007).

**Figure 2:**
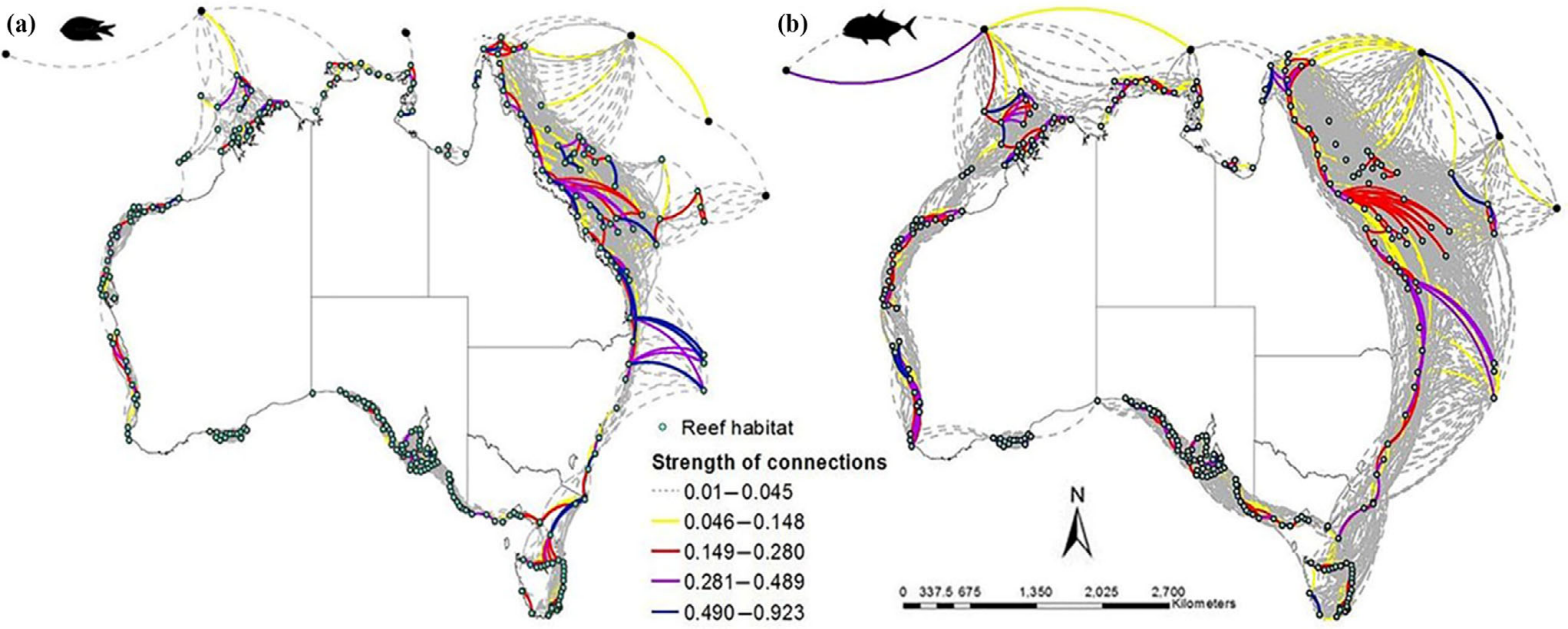
Marine protected area (MPA) connectivity networks for (a) damselfish (22‐day pelagic larval duration [PLD], 7‐day competency period) and (b) trevally (40‐day PLD, 26‐day competency period) derived from the final connectivity matrix (light blue dots, reef; multi‐colored arcs, ecological connectivity; black dots at northern end of study area, reef outside Australia's exclusive economic zone not included in the local scale and MPA analysis; directionality of connectivity, arcs followed in a clockwise direction). Strength of connectivity depicted using the relative inflow matrix.

We used network theory metrics to map where and to what extent the MPAs in the system function as a network (objective 3). Each individual MPA in the system was represented as a node and the movement of larvae (ecological connectivity) as links (Fig. 2). All metrics were calculated using the igraph package in R (Csardi & Nepusz 2006). We first identified which habitat patches were connected (and which were disconnected) by identifying unique components in each species’ network. A component is defined as a group of nodes in which all nodes are connected through dispersal links; nodes in 2 distinct components are therefore by definition not connected (Minor & Urban 2007). The number of unique components in a network provides a measure of the natural fragmentation of the system, with each potentially representing ecologically distinct metapopulations (Minor & Urban 2007; Treml et al. 2008). The proportion of MPAs per component that was linked directly through dispersal was used to represent the degree to which collections of MPAs were functionally connected. This proportion gave an unbiased geographic view of the level to which existing MPAs could function as an ecological network.

## Results

### Quantifying Multi‐species Connectivity

Connectivity varied greatly, depending on the larval dispersal phenotype (Table 1 & Fig. 2). As PLD and precompetency period increased from short to long range dispersers, connectivity also increased across all ecoregions. Unsurprisingly, more numerous and stronger connections were available to species with greater dispersal capacity, such as the wrasse and trevally taxa, that had a maximum PLD of 30 and 40 days, respectively (Fig. 2b & Table 1). Damselfish and trevally taxa had limited connectivity in the tropical north along the Northern Territory and Queensland borders (Fig. 2).

### Local Scale Connectivity Protected in MPAs

**Figure 3:**
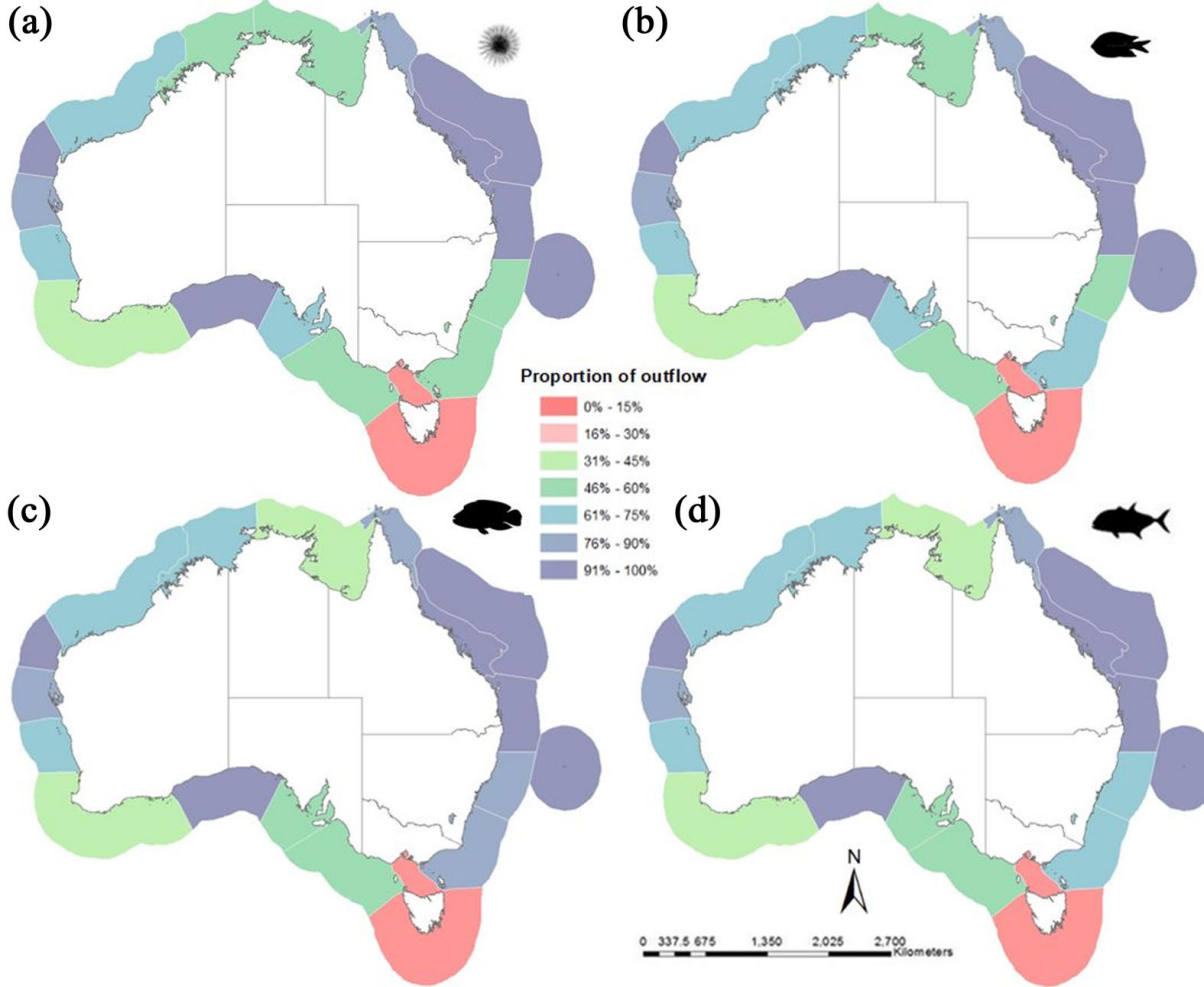
Proportion of the total source strength (i.e., outflow) protected per ecoregion for each modeled phenotype: (a) urchin, (b) damselfish, (c) wrasse, and (d) trevally. High values indicate the site serves as a strong source to downstream areas.

The relative level of source strength and rescue potential protected in MPAs varied considerably across the phenotypes (Supporting Information [Fig. S2.1] & Fig. 3). Although there were some outliers, there was a strong positive correlation (*r_s_* = 0.88) between relative levels of source strength and rescue potential per ecoregion. This is likely because both metrics identified habitats that were strongly connected to and from surrounding habitat patches. The variation in geographic patterns between dispersal phenotypes was a result of the interplay between ocean currents, biological traits, and the distribution of habitat. Ecoregions surrounding the Great Barrier Reef, as well as the Ningaloo and Great Australian Bight ecoregions, protected source strength and rescue potential well across all model phenotypes because they contained large amounts of dense reef habitat with high levels of protection in place. Across all 4 phenotypes, the Bassian ecoregion, surrounding Victoria and Tasmania, consistently had the lowest proportions (<15%) of larval connectivity protected, suggesting many larvae did not reach protected reefs and that the rescue potential for these MPAs was low (Supporting Information [Fig. S2.1] & Fig. 3). Relative to the rest of Australia's ecoregions, the Bassian ecoregion has very low levels of reef habitat protected (3%) as well as fewer and smaller MPAs distributed across the seascape. With respect to the dispersal phenotypes explored, the highest proportion of rescue potential and source strength protected in MPAs was found with the long range dispersers. Furthermore, the proportion of larval outflow protected in MPAs was consistently higher than the proportion of larvae settling from protected upstream sources for all phenotypes. In other words, existing MPAs functioned well as sources of larvae but may also be vulnerable to disturbance due to their low rescue potential from other protected sites (Supporting Information [Fig. S2.1] & Fig. 3).

### Functional MPA Network Connectivity

**Figure 4:**
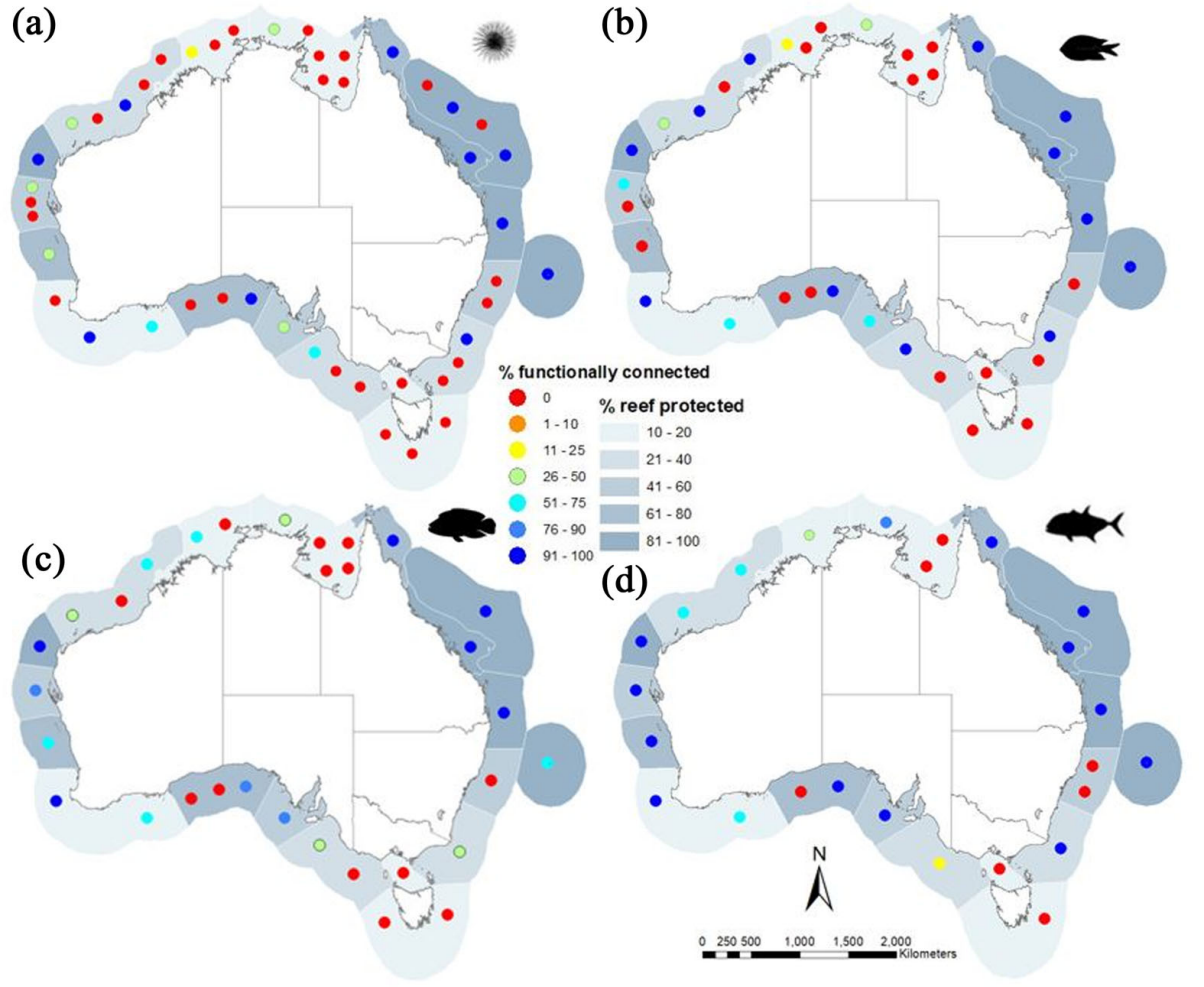
Number of components and the percentage of functionally connected marine protected areas (MPAs) per component for (a) urchin, (b) damselfish, (c) wrasse, and (d) trevally. Each colored dot represents an individual component in an ecoregion, and its color represents the percentage of functionally connected MPAs in that component.

The number of components across ecoregions was negatively correlated with dispersal potential (Table 2), as implied by the biological parameters (Table 1). These results suggest a more fractured network of habitat and lower connectivity for taxa with limited dispersal (e.g., shorter PLDs, limited competency), such as urchins and damselfish, and more cohesion and greater connectivity for taxa with greater dispersal potential such as wrasse and trevally taxa. For example, the median number of components for the urchin across all ecoregions was 3, whereas the trevally had a median of one component per ecoregion. A substantial difference was seen in 4 ecoregions, where the number of components differed by 50% or more between the urchin and trevally model (Table 2). This had significant implications for the proportion of MPAs considered functionally connected. In Australia, the Great Barrier Reef and Ningaloo ecoregions are clear exceptions, and the only ecoregions where MPAs were functioning as a genuine network. In these ecoregions >75% of MPAs were functionally connected, regardless of the dispersal phenotype (Fig. 4). The trevally model, which represented the greatest dispersal potential, had MPAs forming functional networks in 16 of the 25 components analyzed (Fig. 4). The 4 ecoregions that had few functioning MPA networks for the trevally were located in the tropical north around the Northern Territory, the temperate waters around New South Wales, Victoria, and Tasmania, and on the border of South Australia and Western Australia. Of all the MPAs analysed (approximately 2.7 million km^2^ across 18 total ecoregions), outside 2 exceptional ecoregions (Great Barrier Reef & Ningaloo), <50% were considered to belong to a functionally connected protected area network. The total area of MPAs that was functionally connected ranged from 46% for an urchin phenotype to 80% for a trevally phenotype (37 to 65% when the Great Barrier Reef and Ningaloo Reef were excluded).

**Table 2:** Ecoregions surrounding Australia, details on their reefs, and number of components (independent ecological networks) per species

	Ecoregion				Number of components			
Ecoregion	Location on Figure 1	No. of reef patches	No. of protected patches	Area of reef habitat (km^2^)	Urchin	Damselfish	Wrasse	Trevally
Arnhem Coast to Gulf of Carpentaria	1	18	7	9792	6	5	5	3
Bassian	9	34	5	11,008	5	3	3	2
Bonaparte Coast	18	21	11	14,144	3	3	2	1
Cape Howe	8	9	2	3328	3	2	1	1
Central and Southern Great Barrier Reef	4	17	17	120,576	1	1	1	1
Coral Sea	3	19	19	19,904	4	1	1	1
Exmouth to Broome	17	25	10	23,680	5	4	3	2
Great Australian Bight	12	10	10	5248	3	3	3	2
Houtman	14	7	3	5952	1	1	1	1
Leeuwin	13	18	6	6912	3	2	2	2
Lord Howe and Norfolk Islands	6	3	3	768	1	1	1	1
Manning‐Hawkesbury	7	3	1	1920	2	1	1	2
Ningaloo	16	4	4	3648	1	1	1	1
Shark Bay	15	9	5	3904	3	2	1	1
South Australian Gulfs	11	41	26	17,472	2	1	1	1
Torres Strait ‐ Northern Great Barrier Reef	2	12	8	79,616	1	1	1	1
Tweed‐Moreton	5	7	7	9920	1	1	1	1
Western Bassian	10	16	6	6016	2	2	2	1
Total					47	35	30	25

## Discussion

Although the global expansion of MPAs over the last few decades has been labeled a conservation success, there are growing concerns that many MPAs have been established without sufficient reference to the distribution of biodiversity (Jantke et al. 2018) or the connectivity of habitat (Schill et al. 2015), creating challenges for their ability to genuinely support the persistence of marine populations. Although often not the primary objective of MPAs, recent studies have highlighted the importance of integrating connectivity via larval dispersal into MPA design and management and that including connectivity as an objective in marine spatial planning has the potential to alter the optimal design or configuration of MPAs (Krueck et al. 2017; Magris et al. 2018). Our results lend strong support to the idea that the majority of MPAs in the Australian system do not function as networks (Fig. 4). As measured by the number of components for each of the different larval phenotypes (Table 2), Australia's EEZ contains numerous ecological networks separated by natural breaks due to larval traits and the distribution of reefs. Depending on the dispersal capacity of the taxa of interest, there may be between 25 and 47 individual ecological networks distributed across all ecoregions (Table 2). Marine protected areas should be configured to account for this natural fragmentation, leveraging functional connections at this level to ensure they make the greatest contribution to connectivity and the associated benefits (e.g., protecting sources and rescue potential). Our results demonstrate the importance of moving beyond the current focus on the size or shape of protected areas to place a greater emphasis on the configuration of MPAs when evaluating current marine protection or planning new MPAs (O'Leary et al. 2018).

Ideally, MPAs would be large enough to support self‐sustaining populations, but also be sufficiently well connected to enable dispersal among sites. This dispersal is critical to the metapopulation dynamics that can buffer against demographic stochasticity and facilitate gene flow to support genetic diversity and adaptive potential (Beger et al. 2014; Magris et al. 2018). The rescue potential of a habitat patch (larval inflow) is crucial to protect against catastrophic events, such as cyclones or disease outbreaks, which can decimate populations across a whole reef that was previously self‐sustaining. If there is little or no connectivity from nearby habitat patches, then that habitat patch will be less likely or far slower to recover after a severe bottleneck. Therefore, sufficient connectivity from outside MPAs as well as inside is important for long‐term population persistence. The flow of larvae from outside habitat patches is even more crucial for habitats where fishing is permitted (Krueck et al. 2017). These fished habitats are likely to produce fewer larvae than no‐take areas due to fewer individuals present and smallbodied individuals producing fewer larvae (Barneche et al. 2018).

Although important habitat can be identified using numerous metrics, such as representation and the distribution of threats, integrating measures of source strength and rescue potential into the design of MPA systems can contribute to effective management for the benefit of biodiversity conservation. For MPA planning, protecting both the source strength and rescue potential should be a priority because both are crucial for population persistence. However, where compromise is necessary, an MPA with high rescue potential of larvae could potentially compensate for fewer larvae flowing into no‐take MPAs because these unfished areas tend to support larger fish (Edgar et al. 2014) that make a significantly higher contribution to reproductive output (Barneche et al. 2018).

Across all phenotypes, connectivity in the Australian EEZ is only well protected in the Great Barrier Reef, Ningaloo Reef (Western Australia), and sections of the South Australian coastline (Fig. 4). For highly dispersive larvae (i.e., wrasse and trevally), connectivity is generally greater and more broadly distributed, and therefore, a greater proportion is protected in MPAs (Figs. 4c‐d). For those species with limited dispersal capacity, the degree of local scale connectivity protected is very limited (Figs. 4a‐b). Results from the urchin and damselfish models revealed that 49–58% of components (independent ecological networks) across all ecoregions have no functional connectivity between the MPAs they contain, with no direct dispersal among these protected sites (Table 2 & Fig. 4). Our results also demonstrated that the MPA configuration for some ecoregions perform poorly for all dispersal phenotypes, and therefore would benefit from additional MPAs to enable them to function as a network without relying on unprotected patches. However, a focus on area protected alone will not achieve a functioning network of MPAs. To successfully integrate connectivity into MPA planning, future MPAs need to be strategically placed to facilitate dispersal with other MPAs. For example, in some ecoregions (i.e., Great Australian Bight, Manning‐Hawkesbury), protecting 51–72% of reef habitat could still result in components with little to no connectivity among MPAs, depending on which reefs are protected. In contrast, ecoregions with comparatively less habitat protected (20–38%) could achieve >50% of MPAs functionally connected in natural networks (components) for the majority of species (i.e., Leeuwin, Cape Howe) (Table 2 & Figs. 1 & 4).

If our definition of *functionally connected* was relaxed to allow MPAs to be considered connected if dispersal links flow through unprotected sites that also provide habitat (i.e., allowing for habitat stepping stones between protected areas), the results could change significantly. This approach could be used to identify candidate sites for future protection (i.e., the stepping stones) to augment existing MPAs in an effort to strategically expand protected area networks. We identified several ecoregions around Australia that could benefit from this approach (i.e., Bassian, Arnhem Coast to Gulf of Carpentaria, & Bonaparte Coast) due to few reefs being protected and poor connectivity among protected sites. Future MPA planning could also benefit from research exploring the implications of accounting for higher reproductive output from MPAs with larger size classes of target species and more individuals (Edgar et al. 2014). As previous research suggests that larger females in a population contribute disproportionately to reproductive output than smaller females (Barneche et al. 2018), no‐take marine reserves should be prioritized in future MPA planning and management as a way to potentially replenish fisheries (Roberts et al. 2001).

Larval behavior affects local retention and local scale connectivity of larvae for some taxa (Paris et al. 2007). A challenge of accounting for larval behavior in connectivity models is the evidence, both empirical (Gerlach et al. 2007) and model‐based (Treml et al. 2015), that the impact of behavior on broadscale connectivity is highly variable, dependent on larval biology and seascape context (i.e., where the larvae are), and can be difficult to extrapolate beyond local scales (i.e., meters). Although most larvae have behavior, incorporating this information into our dispersal model was difficult due to a lack of empirical data and confidence in how to parameterize these dynamic behavioral traits (e.g., when does behavior develop, how strong is swimming, what are the vertical swimming strategies, what are the sensing capacities, etc.?). While we elected to model passive larvae, some previous modeling efforts have provided greater resolution by incorporating larval behavior to better understand how swimming (Fiksen et al. 2007) and vertical distribution strategies (Crosbie et al. 2019) influence dispersal.

Our model was designed to be inclusive of a wide range of species and accommodate different dispersal strategies. Therefore, our findings are transferrable to other similar species and our approach can be used to understand connectivity in any existing or proposed system of MPAs. Our results suggest that including connectivity concepts in MPA design would increase the capacity to achieve conservation objectives such as species persistence. Furthermore, these results highlight the need to evaluate the underlying natural connectivity of a study system prior to implementing new MPAs and to consider which species are likely to benefit from the protection of additional areas. It also highlights the risk of assuming that a collection of MPAs in a system will automatically function as a network. As estimates of ecological connectivity from dispersal modeling become more feasible and are increasingly available, MPA planners may benefit by explicitly incorporating connectivity objectives alongside other conservation goals, particularly from a multi‐species perspective, to better ensure species’ persistence.

## Supporting information

Comparison tables of life history parameter PLD for similar taxa to 4 dispersal phenotypes (Appendix S1) is available online. The authors are solely responsible for the content and functionality of these materials. Queries (other than absence of the material) should be directed to the corresponding author.

Supplementary Material
