# Supplementary material for: Assessing the current state of ecological connectivity in a large marine protected area system
Source: Conserv Biol. 2020 Sep 5;35(2):699–710. doi: 10.1111/cobi.13580 (PMC8048790; doi:10.1111/cobi.13580)
Supplement: Supplementary file 1 — Comparison tables of life history parameter PLD for similar taxa to 4 dispersal phenotypes (Appendix S1) is available online. The authors are solely responsible for the content and functionality of these materials. Queries (other than absence of the material) should be directed to the corresponding author. [file COBI-35-699-s002.docx]

**Supporting Information**

**Appendix S1.** **Comparison tables of life history parameter PLD for similar taxa to 4 dispersal phenotypes**

**Table S1.1** Comparison of similar taxa PLD (in days) to modelled species *urchin*

| FAMILY | SCIENTIFIC NAME | PLD | SOURCE |
| --- | --- | --- | --- |
| Ophiochitonidae | *Ophionereis schayeri* | 7 | Falkner & Byrne 2003 |
| Anthozoa | *Alcyonium siderium* | 4.5 | Shanks 2009 |
| Cladorhizidae | *Stylopsus spp.* | 2.5 | Ayling 1980 |
| Onchidorididae | *Adalaria proxima* | 2 | Shanks 2009 |
| Vermetidae | *Dendropoma corallinaceum* | 3.5 | Hughes 1978 |
| Acanthasteridae | *Acanthaster planci* | 14 | Moran et al. 1992 |
| Echinometridae | *Heliocidaris erythrogramma* | 5 | Williams & Anderson 1975 |
| Balanidae | *Balanus glandula* | 14 | Schwindt 2007 |
| Muricidae | *Rapana venos* | 14 | Shanks 2009 |
|  |  |  |  |
|  |  |  |  |
|  |  |  |  |

**Table S1.2** Comparison of similar taxa PLD (in days) to modelled species *damselfish*

| FAMILY | SCIENTIFIC NAME | PLD | SOURCE |
| --- | --- | --- | --- |
| Pomacentridae | *Chromis cyanea* | 29 | Wellington & Victor 1989 |
| Pomacentridae | *Centropyge tibicen* | 30 | Wellington & Victor 1989 |
| Pomacentridae | *Plectroglyphidodon sindonis* | 30 | Wellington & Victor 1989 |
| Pomacentridae | *Chromis alpha* | 30 | Wellington & Victor 1989 |
| Pomacentridae | *Chromis hanui* | 27 | Wellington & Victor 1989 |
| Pomacentridae | *Chromis insolata* | 20 | Wellington & Victor 1989 |
| Pomacentridae | *Dascyllus reticulatus* | 20 | Wellington & Victor 1989 |
| Pomacentridae | *Chromis xanthura* | 26 | Wellington & Victor 1989 |
| Pomacentridae | *Chromis traceyi* | 23 | Wellington & Victor 1989 |
| Pomacentridae | *Stegastes lividus* | 25 | Wellington & Victor 1989 |
|  |  |  |  |
|  |  |  |  |
|  |  |  |  |

**Table S1.3** Comparison of similar taxa PLD (in days) to modelled species *wrasse*

| FAMILY | SCIENTIFIC NAME | PLD | SOURCE |
| --- | --- | --- | --- |
| Labridae | *Halichoeres semicinctus* | 29 | Victor & Wellington 2000 |
| Labridae | *Macropharyngodon meleagris* | 30 | Brothers & Thresher 1985 |
| Labridae | *Coris batuensis* | 30 | Froese & Pauly 2006 |
| Labridae | *Cirrhilabrus temminki* | 30 | Brothers & Thresher 1985 |
| Labridae | *Bodianus mesothorax* | 30.3 | Victor 1986 |
| Labridae | *Halichoeres nicholsi* | 30.4 | Victor & Wellington 2000 |
| Labridae | *Epibulus insidiator* | 30.4 | Victor 1986 |
| Labridae | *Labropsis xanthonota* | 30.5 | Victor 1986 |
| Labridae | *Halichoeres maculipinna* | 30.5 | Schultz & Cowen 1994 |
| Acanthuridae | *Acanthurus nigrofuscus* | 31 | Wilson & McCormick 1999 |
|  |  |  |  |
|  |  |  |  |
|  |  |  |  |

**Table S1.4** Comparison of similar taxa PLD (in days) to modelled species *trevally*

| FAMILY | SCIENTIFIC NAME | PLD | SOURCE |
| --- | --- | --- | --- |
| Carangidae | *Caranx melampygus* | 30 | Kim et al. 2001 |
| Polynemidae | *Polydactylus sexfilis* | 30 | Kim et al. 2001 |
| Carangidae | *Caranx ignobilis* | 40 | Sudekum et al. 1991 |
| Scorpaenidae | *Pterois volitans* | 40 | Shanks 2009 |
| Carangidae | *Caranx ignobilis* | 40 | Leis & Carson-Ewart 2001 |
| Carangidae | *Pseudocaranx dentex* | 45 | Paxton et al. 1989 |
| Mullidae | *Mulloidichthys flavolineatus* | 45 | Longenecker & Langston 2008 |
| Mullidae | *Parupeneus porphyreus* | 47 | Longenecker & Langston 2008 |
|  |  |  |  |

**Literature cited**

Ayling AL. 1980. Patterns of sexuality, asexual reproduction and recruitment in some subtidal marine demospongiae. The Biological Bulletin **158**:271–282.

Brothers EB, Thresher RE. 1985. Pelagic duration, dispersal, and the distribution of Indo-Pacific coral reef fishes. In: Reaka, M. (ed.) The ecology of coral reefs. U.S. Department of Commerce, Washington, D.C., 53 -69.

Falkner I, Byrne M. 2003. Reproduction of *Ophiactis resiliens* (Echinodermata: Ophiuroidea) in New South Wales with observations on recruitment. Marine Biology **143**:459-466.

Froese R, Pauly D. 2006. FishBase. Available at http://www.fishbase.org [accessed August 2018].

Hughes RN. 1978. The biology of *Dendropma corallinaceum* and *Serpulorbis natalensis*, two South African vermetid gastropods. Zoological Journal of the Linnean Society **64**:111–127.

Kim BG, Divakaran S, Brown CL, Ostrowski AC. 2001. Comparative digestive enzyme ontogeny in two marine larval fishes: Pacific threadfin (*Polydactylus sexfilis*) and bluefin trevally (*Caranx melampygus*). Fish Physiology and Biochemistry **24**:225-241.

Leis JM, Carson-Ewart BM. 2001. Behavioural differences in pelagic larvae of four species of coral- reef fishes between two environments: ocean and atoll lagoon. Coral Reefs **19**:247−257.

Longenecker K, Langston R. 2008. Life history compendium of exploited Hawaiian fishes. Fisheries Local Action Strategy and Division of Aquatic Resources.

Moran PJ, De’ath G, Baker VJ, Bass DK, Chistie CA, Miller IR, Miller-Smith BA, Thompson AA. 1992. Pattern of outbreaks of crown-of thorns starfish (*Acanthaster planci L*.) along the Great Barrier Reef since 1966. Australian Journal of Marine and Freshwater Research **43**:555–568.

Paxton JR, Hoese DF, Allen GR, Hanley JE. 1989. Zoological Catalogue of Australia. Pisces. Petromyzontidae to Carangidae. Australian Government Publishing Service: Canberra. Zoological Catalogue of Australia **7**:1–665.

Schultz ET, Cowen RK. 1994. Recruitment of coral-reef fishes to Bermuda: local retention or long- distance transport? Marine Ecology Progress Series **109**:15–28.

Schwindt E. 2007. The invasion of the acorn barnacle *Balanus glandula* in the south-western Atlantic 40 years later. Journal of the Marine Biological Association of the United Kingdom **87**:1219–1225.

Shanks AL. 2009. Pelagic larval duration and dispersal distance revisited. The Biological Bulletin **216**:373-385.

Sudekum AE, Parrish JD, Radtke RL, Ralston S. 1991. Life history and ecology of large jacks in undisturbed, shallow, oceanic communities. Fishery Bulletin **89**:493−513.

Victor BC. 1986. Duration of the planktonic larval stage of one hundred species of Pacific and Atlantic wrasses (family Labridae). Marine Biology **90**:317–326.

Victor BC, Wellington GM. 2000. Endemism and the pelagic larval duration of reef fishes in the eastern Pacific Ocean. Marine Ecology Progress Series **205**:241-248.

Wellington GM, Victor BC. 1989. Planktonic larval duration of one hundred species of Pacific and Atlantic damselfishes (Pomacentridae). Marine Biology **101**:557–567.

Williams DC, Anderson DT. 1975. The reproductive system, embryonic development, larval development and metamorphosis of the sea urchin *Heliocidaris erythrogramma* (Val.) (Echinoidea : Echinometridae). Australian Journal of Zoology **23**:371-403.

Wilson DT, McCormick MI. 1999. Microstructure of settlement-marks in the otoliths of tropical reef fishes. Marine Biology **134**:29–41.
