# Supplementary material for: Assessing the current state of ecological connectivity in a large marine protected area system
Source: Conserv Biol. 2020 Sep 5;35(2):699–710. doi: 10.1111/cobi.13580 (PMC8048790; doi:10.1111/cobi.13580)
Supplement: Supplementary file 2 — Supplementary Material [file COBI-35-699-s001.docx]

**Supporting Information**

**Appendix S2. Local-scale connectivity protected within MPAs – proportion of inflow**

**
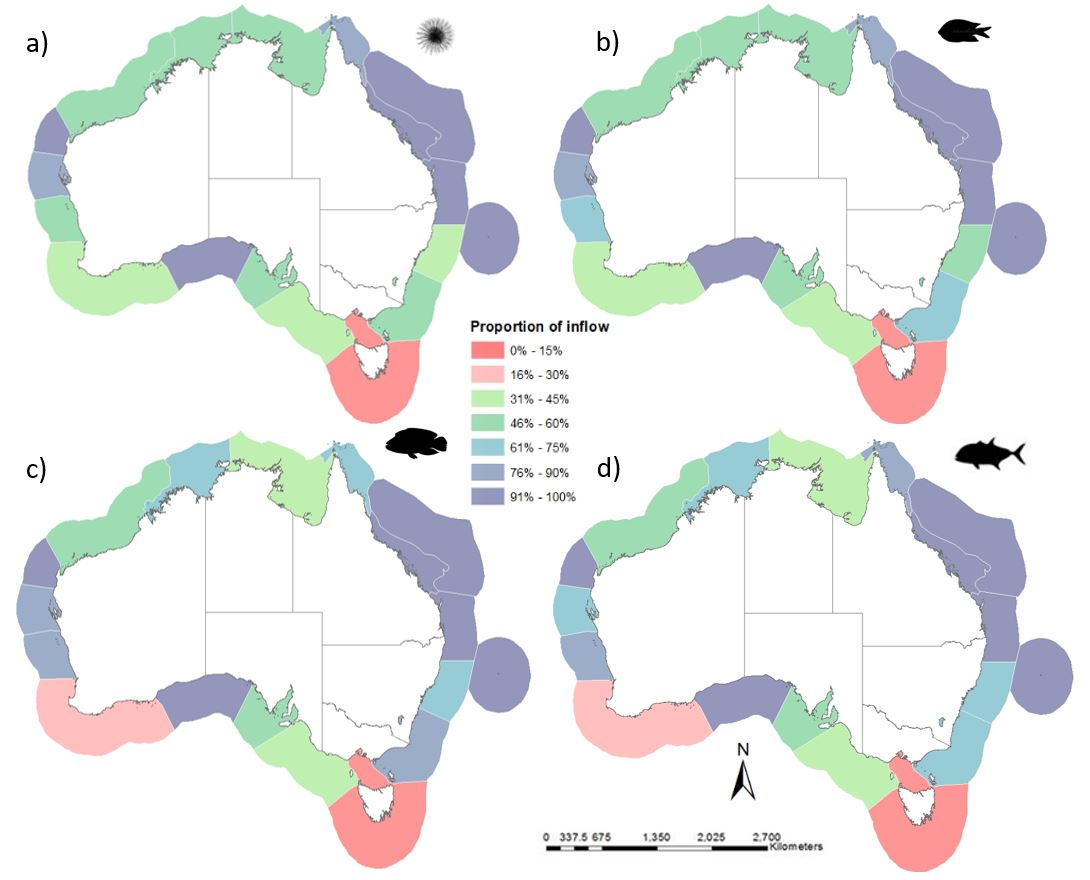
**

**Figure S2.1.** Proportion of the total rescue potential (i.e., inflow) protected per ecoregion for each modeled phenotype: (a) urchin, (b) damselfish, (c) wrasse, and (d) trevally. The higher the value, the higher the immigration potential (i.e., a site benefiting from strong upstream sources).
